# Supplementary material for: Asymmetric gating of a human hetero-pentameric glycine receptor
Source: Nat Commun. 2023 Oct 11;14:6377. doi: 10.1038/s41467-023-42051-6 (PMC10567788; doi:10.1038/s41467-023-42051-6)
Supplement: Supplementary file 1 — Supplementary Information [file 41467_2023_42051_MOESM1_ESM.pdf]

Supplementary Information

Asymmetric gating of a human hetero-pentameric glycine receptor

Xiaofen Liu[1], Weiwei Wang[1*]

[1]Departments of Biophysics, University of Texas Southwestern Medical Center, Dallas, TX, USA.

*Correspondence: Weiwei.Wang@UTSouthwestern.edu

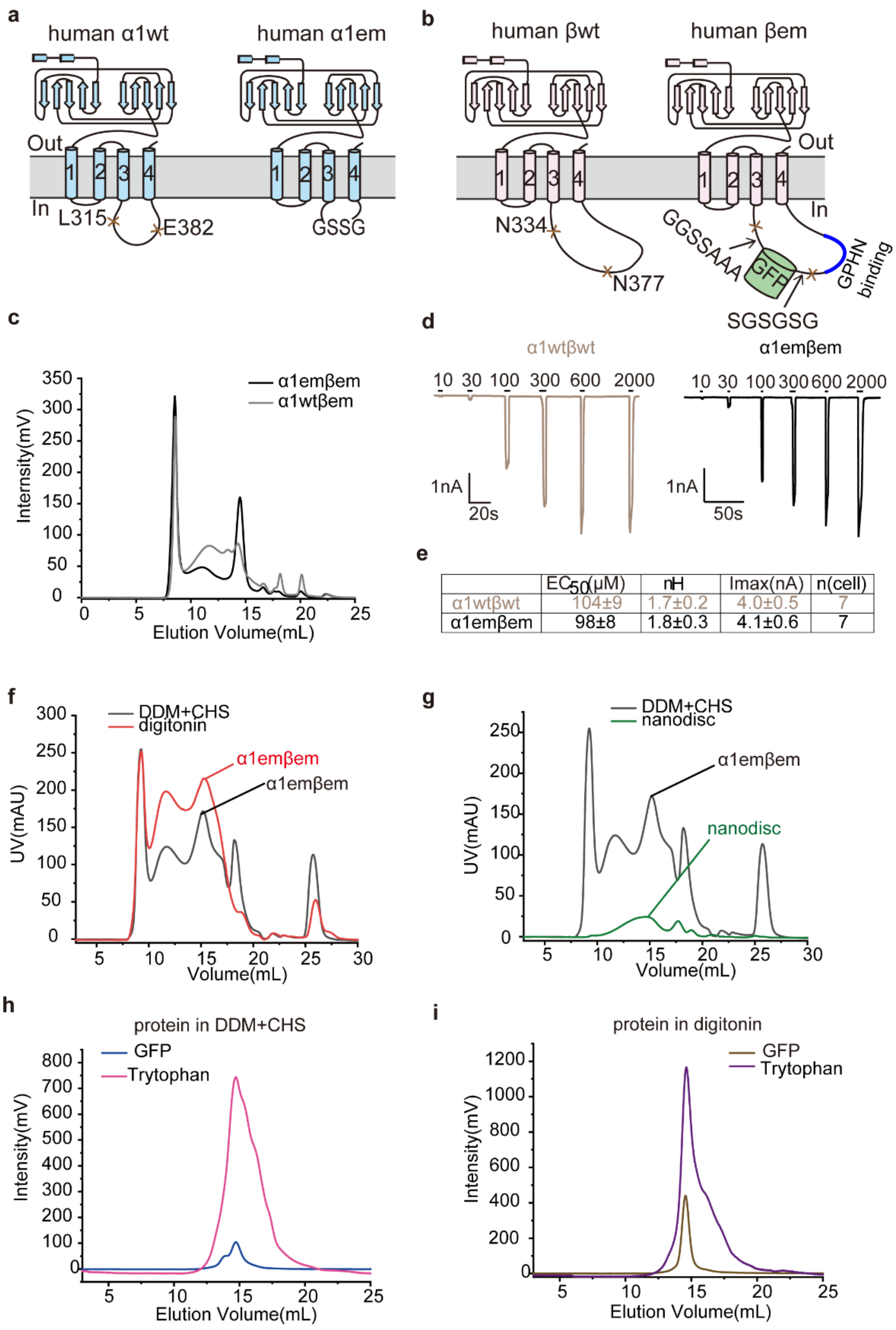

	EC_{50}(μM)	nH	Imax(nA)	n(cell)
α1wtβwt	104±9	1.7±0.2	4.0±0.5	7
α1emβem	98±8	1.8±0.3	4.1±0.6	7

Supplementary Figure 1 | The topology of α1 and β GlyRs constructs and whole cell voltage-clamp electrophysiology. (**a**)The topology diagrams of human α1wt and α1em. (**b**)The topology

diagrams of human βwt and βem. (**c**) FSEC of α1wtβem and α1emβem expressed at 1:3 of α1:β virus ratio. The trace represented GFP fluorescence (excitation 480nm, emission 515nm) of elution from a Superose6 increase 10/300 column. Source data are provided as a Source Data file. (**d**) Representative whole cell voltage-clamp electrophysiology recordings of glycine dose response for Fig.1**a**. (**e**) EC_{50}, *nH* (Hill coefficient), *Imax*, and *n* (cells) are listed. (**f**) Representative size-exclusion chromatography profile of the α1βGlyRs in DDM+CHS and Digitonin. Source data are provided as a Source Data file. (**g**) Representative size-exclusion chromatography profile of theα1 GlyRs βin DDM+CHS and reconstituted in nanodiscs. Source data are provided as a Source Data file. (**h**) FSEC of purified α1β GlyRs in DDM + CHS. Red and blue traces represented the tryptophan signal (excitation 280nm, emission 340nm) and GFP fluorescence (excitation 480nm, emission 515nm), respectively. Source data are provided as a Source Data file. (**i**) FSEC of purified α1β GlyRs in digitonin. Purple and brown traces represented the tryptophan signal (excitation: 280nm, emission 340nm) and GFP fluorescence (excitation 480nm, emission 515nm), respectively. Source data are provided as a Source Data file.

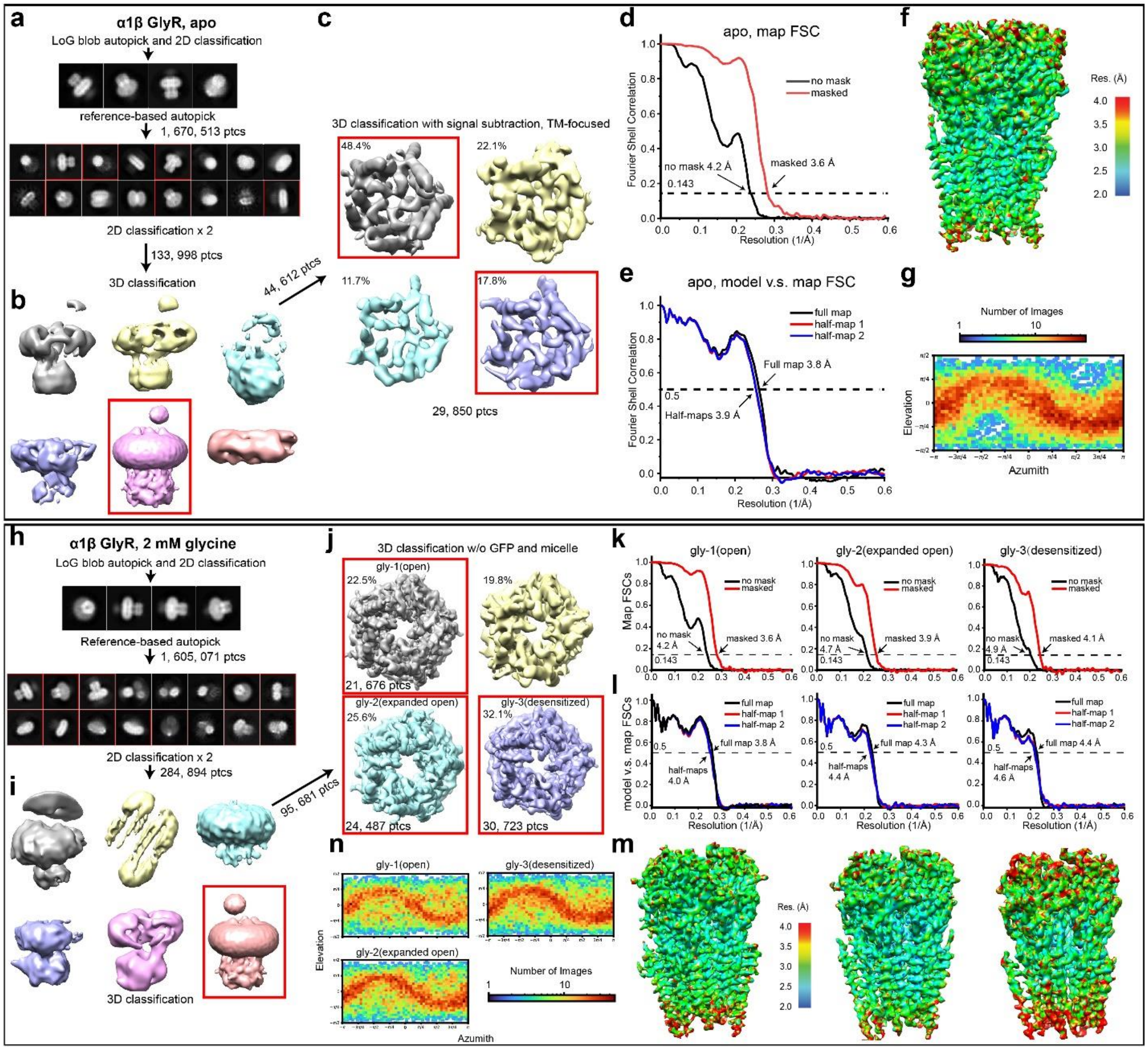

Supplementary Figure 2 | Overview cryo-EM data processing.

(**a**-**f**) The apo state of α1β GlyR. (**a**,**b**) Selected (red boxes) 2D and 3D classes average for the apo state of α1β GlyR. (**c**) 4 classes resulting from 3D classification. The percentages of particles in each class were showed. The two good classes were combined for further refined. (**d**) The gold standard FSC curves from the final map of the apo state. (**e**) FSC between the atomic model and half maps/full map. Resolution at FSC of 0.5 was indicated. (**f**)Local resolution maps for the apo state. (**g**) A representation of the angular distribution of particles used in the final reconstruction

for apo state. (**h**-**n**) The gly-1(open), gly-2(expanded open) and gly-3(desensitized) states of α1β GlyR. **(h**, **i**) Selected (red boxes) 2D and 3D classes average for the glycine-bound α1β GlyR. (**j**) 4 classes resulting from 3D classification. The percentages of particles in each class are shown. The 3 good classes in boxes were selected for further refined. (**k**) Gold standard FSC curves of the final maps of the open state, expanded open state, desensitized state. (**l**)FSC between the atomic model and half maps/full map. Resolution at FSC of 0.5 was indicated. (**m**) Local resolution maps for the gly-1(open) state, gly-2 (expanded open) state and gly-3(desensitized) states. Local resolutions are calculated using ResMap. (**n**)A representation of the angular distribution of particles used in the final reconstruction for gly-1(open), gly-2(expanded open) and gly-3(desensitized) states.

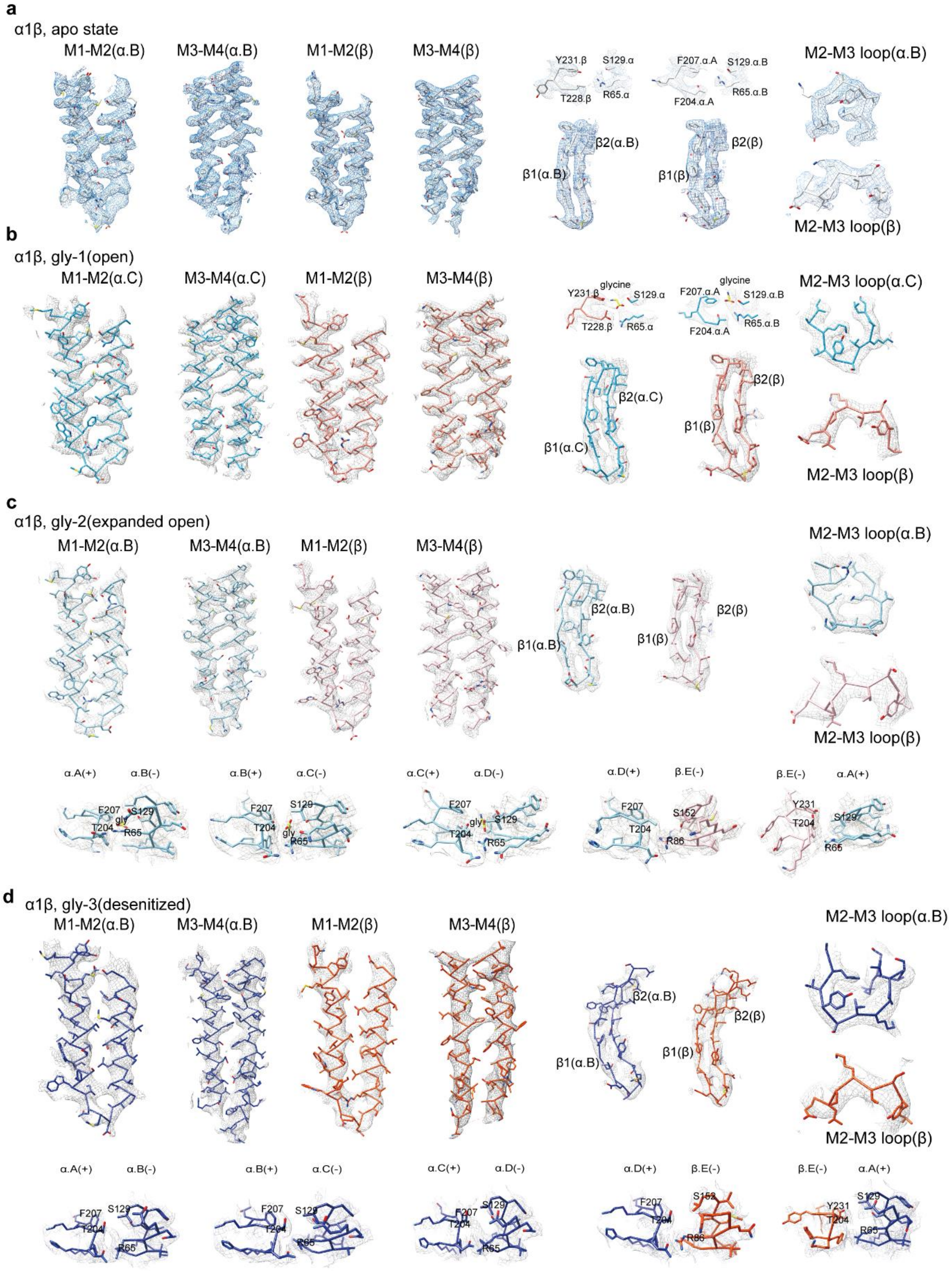

a
α1β, apo state
M1-M2(α.B)
M3-M4(α.B)
M1-M2(β)
M3-M4(β)
Y231.β
S129.α
T228.β
R65.α
F207.α.A
S129.α.B
R65.α.B
F204.α.A
M2-M3 loop(α.B)
β2(α.B)
β1(α.B)
β2(β)
β1(β)
M2-M3 loop(β)
b
α1β, gly-1(open)
M1-M2(α.C)
M3-M4(α.C)
M1-M2(β)
M3-M4(β)
glycine
Y231.β
S129.α
T228.β
R65.α
glycine
F207.α.A
S129.α.B
F204.α.A
R65.α.B
M2-M3 loop(α.C)
β2(α.C)
β1(α.C)
β2(β)
β1(β)
M2-M3 loop(β)
c
α1β, gly-2(expanded open)
M1-M2(α.B)
M3-M4(α.B)
M1-M2(β)
M3-M4(β)
M2-M3 loop(α.B)
β2(α.B)
β1(α.B)
β2(β)
β1(β)
M2-M3 loop(β)
α.A(+)
α.B(-)
α.B(+)
α.C(-)
α.C(+)
α.D(-)
α.D(+)
β.E(-)
β.E(-)
α.A(+)
d
α1β, gly-3(desenitized)
M1-M2(α.B)
M3-M4(α.B)
M1-M2(β)
M3-M4(β)
M2-M3 loop(α.B)
β2(α.B)
β1(α.B)
β2(β)
β1(β)
M2-M3 loop(β)
α.A(+)
α.B(-)
α.B(+)
α.C(-)
α.C(+)
α.D(-)
α.D(+)
β.E(-)
β.E(-)
α.A(+)

Supplementary Figure 3 | cryo-EM densities for selected regions of α1β GlyR.

For apo state (**a**) and gly-1(open) state (**b**), left to right, M1-M4 helices of the α1 subunit and β subunit, orthosteric pockets at β(+)α(-) and α(+)α(-) interfaces, β1-β1 sheet, and M2-M3 loop of the α1 subunit (variants of blue) and β subunit (variants of orange). For gly-2(expanded open) state (**c**) and gly-3(desensitized) state (**d**), from left to right in the first row, M1-M4 helices of the α1 subunit and β subunit, β1-β1 sheet, and M2-M3 loop of the α1 subunit (variants of blue) and β subunit (variants of orange).From left to right in the second row, orthosteric pockets at β(+)α(-) and α(+)α(-) interfaces of the α1 subunit (variants of blue) and β subunit (variants of orange)

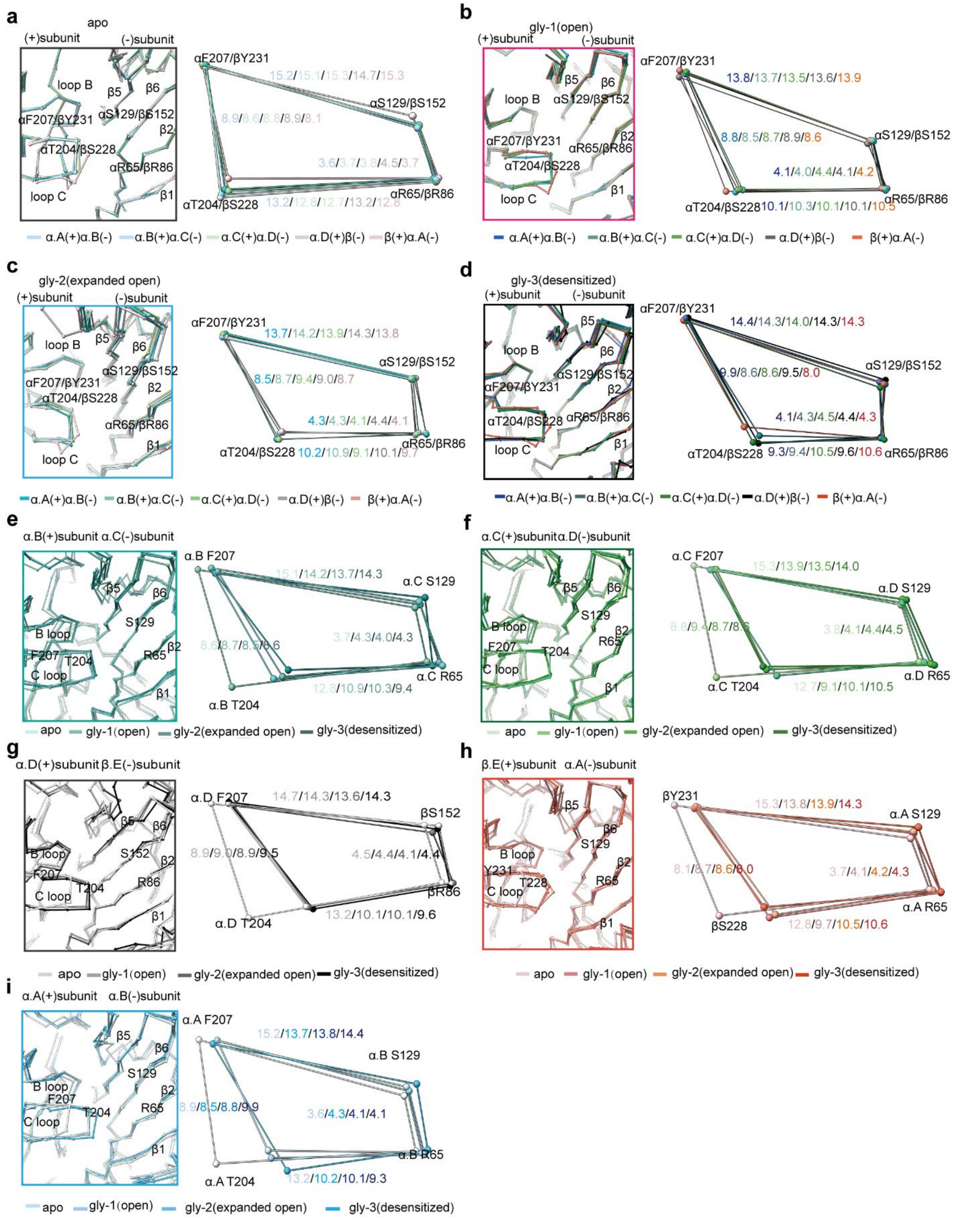

Supplementary Figure 4 | Comparison of the glycine binding pocket.

(a, **b**, **c**, **d)** Superposition of all 5 glycine binding pockets in each of the apo (**a**), gly-1(open) (**b**), gly-2(expanded open) (**c**) and gly-3(desensitized) (**d**) states, with schematic diagram illustrating the relative positions of key residues in (+) and (–) sides shown on the right. (**e**, **f**, **g**, **h**, **i**) Conformational changes of glycine binding pockets at α.A(+)α.B(-) (**e**), α.B(+)α.C(-) (**f**), α.C(+)α.D(-) (**g**), α.D(+)β(-) (**h**), β(+)α.A(-) (**i**) interfaces superimposed across the apo, gly-1(open), gly-2(expanded open) and gly-3(desensitized) states (aligned using ECD). Schematic diagram illustrating the change in distance of key residues are shown on the right. All distances are given in Å.

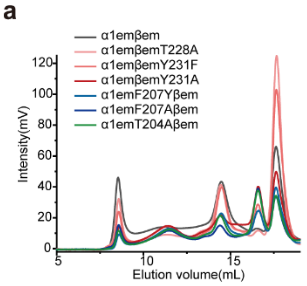

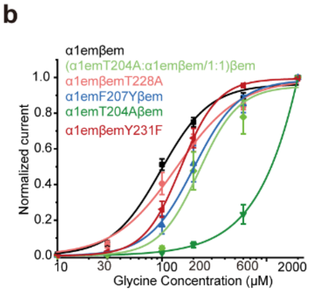

	EC50(μM)	nH	Imax(nA)	n(cell)
α1emβem	98±8	1.8±0.3	4.1±0.6	7
α1emF207Aβem*	715±211	1.3±0.2	0.2±0.02	9
(α1emT207A:α1emβem/1:1)βem	189±9	3.6±0.7	2.8±0.4	8
α1emβemY231A	214±9	2.7±0.3	2.0±0.5	6
α1emβemT228A	137±9	1.4±0.2	4.7±0.8	7
α1emβemY231F	152±6	2.4±0.2	2.6±0.5	10
α1emT204Aβem*	10130±833	1.3±0.6	1.2±0.2	7
(α1emT204A:α1emβem/1:1)βem	266±20	2.3±0.4	2.5±0.2	7
α1emF207Yβem	201±18	2.0±0.3	2.9±0.5	10

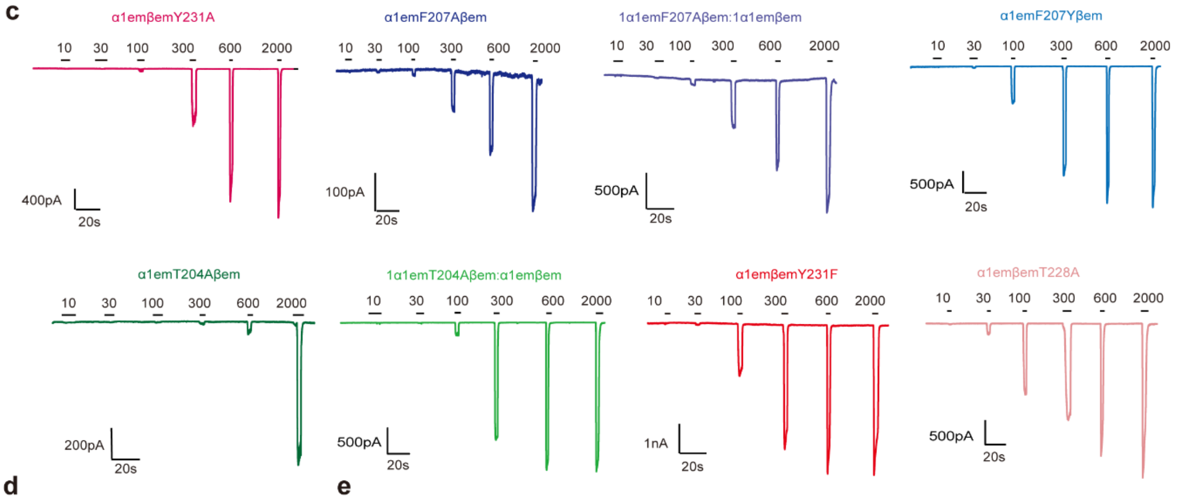

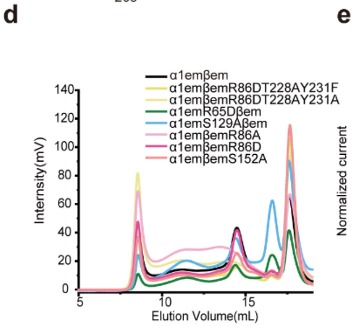

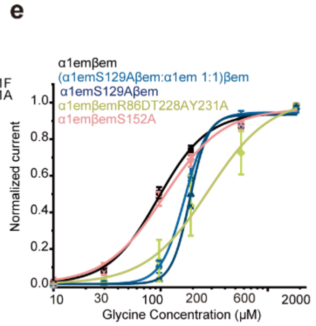

	EC50(μM)	n_H	I_{max}(nA)	n(cell)
α1emβem	98±8	1.8±0.3	4.1±0.6	7
α1emR65Dβem	843±-	0.7±0.1	0.08±0.01	9
1α1emR65Dβem:1α1emβem	187±5	5.0±1	4.3±1	7
1α1emS129Aβem	175±5	5.0±1.2	2.6±0.7	6
α1emS129Aβem:1α1emβem	196±6	3.9±0.4	4.6±0.8	6
α1emβemS152A	111±10	1.6±0.2	4.0±0.8	6
α1emβemR86T	154±6	5.0±0.6	8.7±1.1	7
α1emβemR86TY231A	258±25	1.7±0.2	5.6±0.4	7
α1emβemR86DT228AY231A	293±38	1.5±0.2	5.2±1.0	11
α1emR271Aβem	>1000	1.5±0.8	0.035±0.01	6
α1emR271Eβem	101±9	1.8±0.2	4±0.2	7

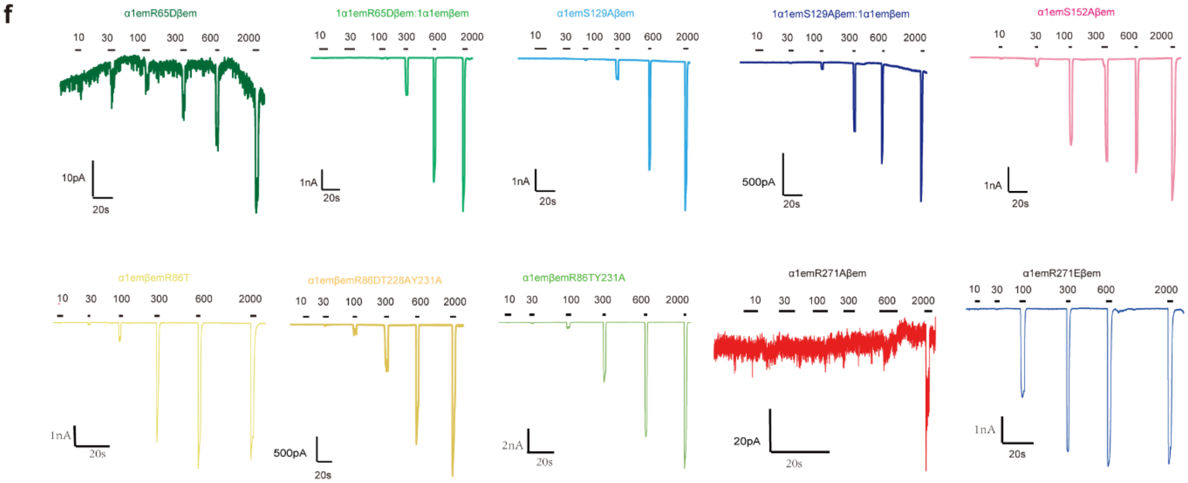

Supplementary Figure 5 | FSCE expression assay and dose response of glycine for glycine binding pocket mutants.

(a) FSEC of α (+) side and β (+) side mutants. All trace represented the GFP signal arising from fusion on the β subunit. Source data are provided as a Source Data file. (**b**) Glycine dose response of (+) side mutants. Source data are provided as a Source Data file. (**c**) Typical complete whole cell voltage-clamp electrophysiology traces of mutants in panel b. (**d**) FSEC of α (-) side and β (+)/β (-) double sides mutants. Source data are provided as a Source Data file. (**e**) Glycine dose response curves of mutants in panel d. Source data are provided as a Source Data file. (**f**)Typical complete whole cell voltage-clamp electrophysiology traces of mutants in panel **e**. Data are represented as mean ± SEM. EC_{50}, *n* (Hill coefficient), *Imax*, and *n* (cells) are listed on the side.

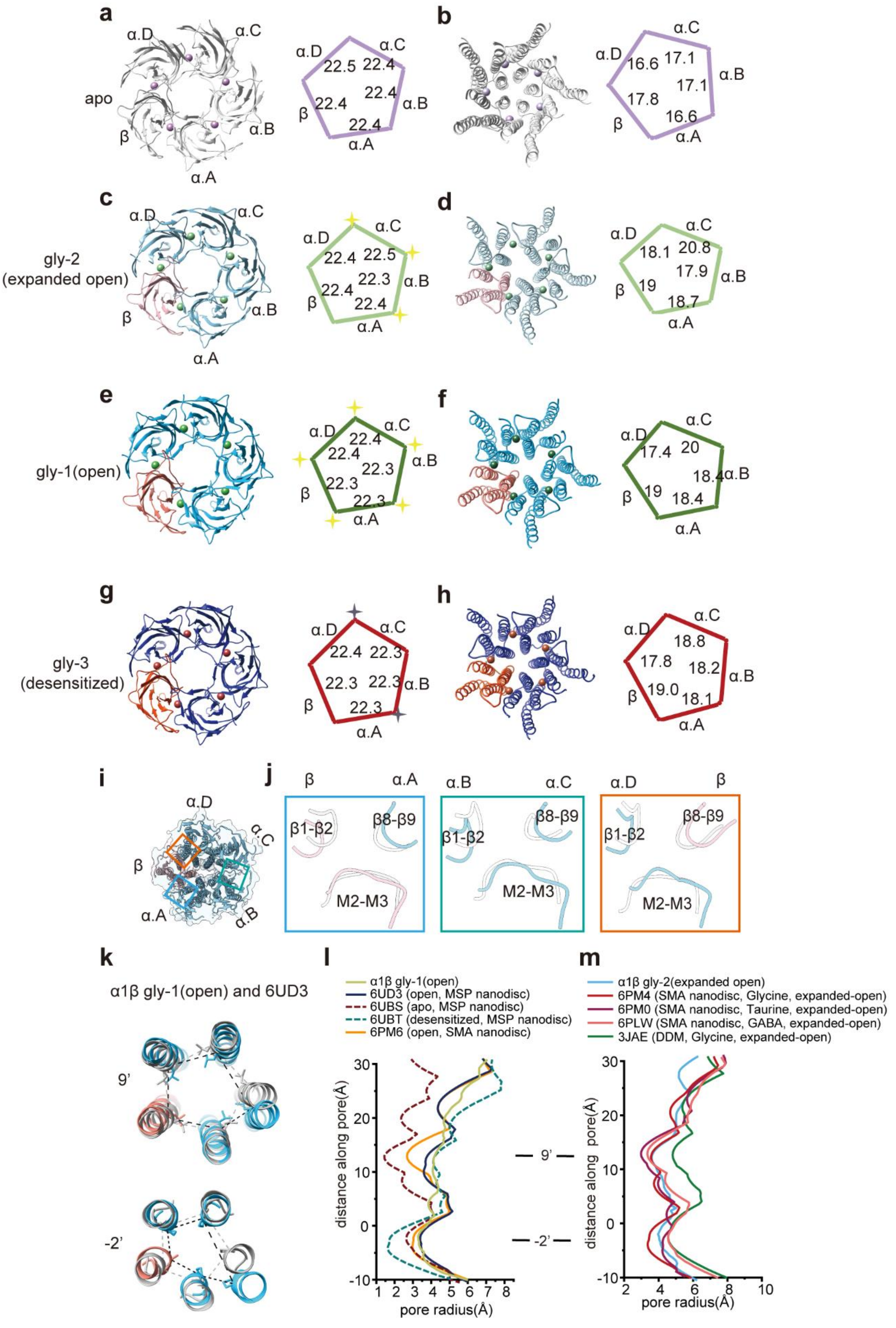

a
α.D
α.C
apo
β
α.B
α.A
22.5
22.4
22.4
22.4
22.4
22.4
b
16.6
17.1
17.1
17.8
16.6
c
gly-2
(expanded open)
22.4
22.5
22.3
22.4
22.4
d
18.1
20.8
17.9
19
18.7
e
gly-1(open)
22.4
22.4
22.3
22.3
22.3
f
17.4
20
18.4
19
18.4
g
gly-3
(desensitized)
22.4
22.3
22.3
22.3
22.3
h
18.8
17.8
18.2
19.0
18.1
i
j
β1-β2
β8-β9
M2-M3
k
α1β gly-1(open) and 6UD3
9'
-2'
l
α1β gly-1(open)
6UD3 (open, MSP nanodisc)
6UBS (apo, MSP nanodisc)
6UBT (desensitized, MSP nanodisc)
6PM6 (open, SMA nanodisc)
distance along pore(Å)
pore radius(Å)
m
α1β gly-2(expanded open)
6PM4 (SMA nanodisc, Glycine, expanded-open)
6PM0 (SMA nanodisc, Taurine, expanded-open)
6PLW (SMA nanodisc, GABA, expanded-open)
3JAE (DDM, Glycine, expanded-open)
distance along pore(Å)
pore radius(Å)

Supplementary Figure 6 | Overall geometry of α1β GlyR throughout the gating cycle and conformational changes at the ECD-TMD interface.

Cartoon representation of ECD (**a, c, e, g**) and TMD (**b, d, f, h**), with diagram on the right showing distances between the centers of mass of two adjacent subunits. (**a**, **b**,) apo state. α: white. β: gray. (**c**, **d**) gly-2(expanded open) state. α: light sky blue. β: pink. (**e**, **f**) gly-1(open) state. α: sky blue. B: salmon; (**g, h**) gly-3(desensitized) state. α: blue. B: tomato. The yellow stars in c panel and e panel represent glycine bound in binding pocket. The grey stars indicate pockets with markedly glycine density. (**i**) Top-down view of ECD- TMD interface. (**j**) Overlay of ECD-TMD interface between apo and gly-1(open) states at β(+) α.A(-) (left), α.B(+)α.C(-) (middle) and α.D(+)β(-) (right) interfaces. All the distances are given in Å. (k) Comparison of cross-sections of M2 helices at residues 9' (top) and -2' (bottom) in the α1β open(α:blue,β:pink) and 6UD3(grey). (**l**) Plot of pore radii calculated by the HOLE program for the α1β gly-1(open) (yellow), 6UD3 (blue), 6UBS (red), 6UBT (green), 6PM6 (orange). Source data are provided as a Source Data file. (**m**) Plot of pore radii calculated by the HOLE program for the α1β gly-2(expanded open) state (light blue), 6PM4 (red), 6PM0(purple), 6PLW(orange) and 3JAE(green). Source data are provided as a Source Data file.

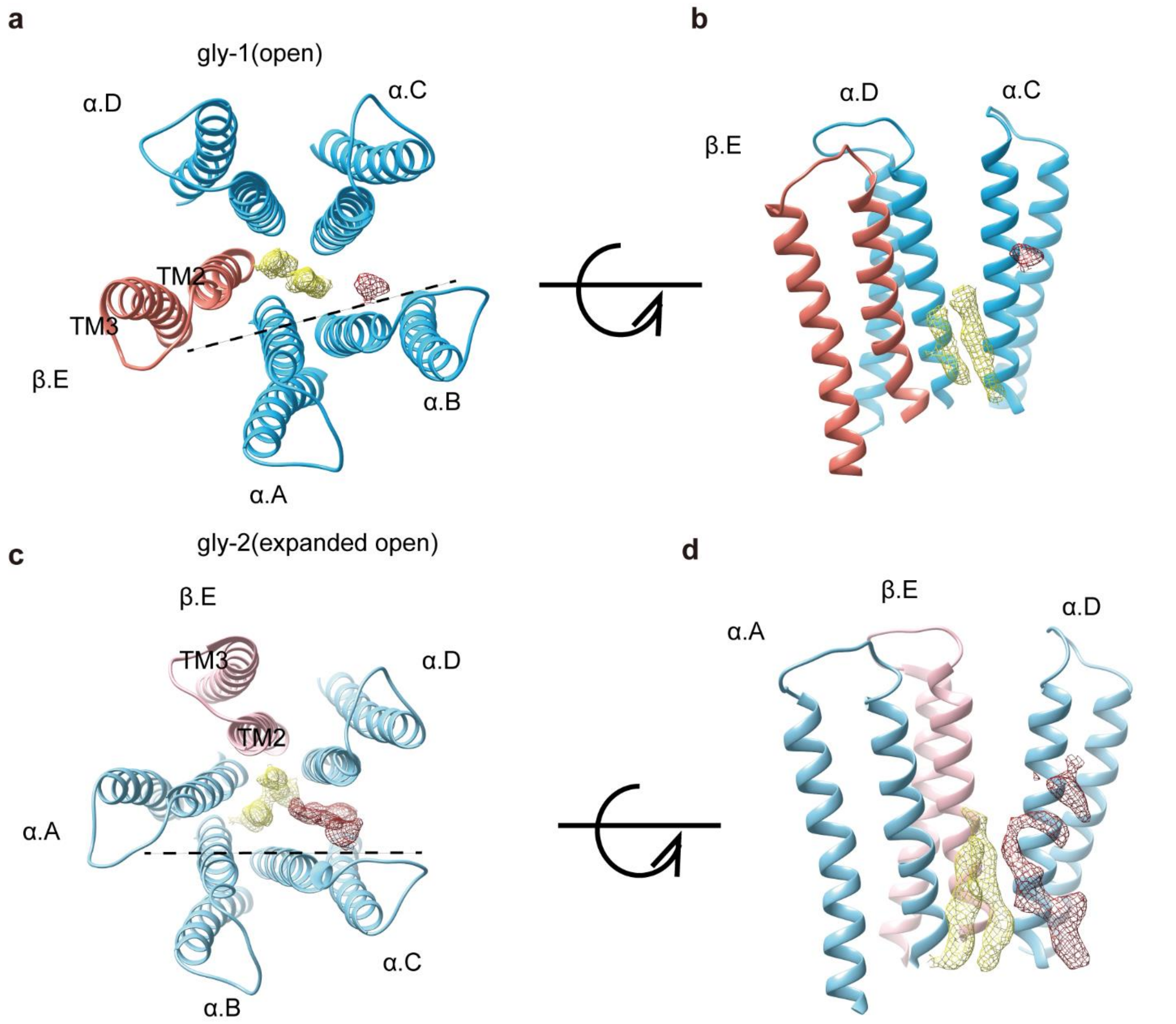

Supplementary Figure 7 | Non-protein densities of in the gly-1(open) and gly-2(expanded open) structures, both contoured at 5 RMSD. Densities at widened subunit interfaces, and those in conduction pathways are colored in red and yellow, respectively. (a) Top-down view and (b) side view of gly-1 (open). (c) Top-down view and (d) side view of gly-2 (expanded open). Clipping planes are indicated for side-views.

Supplementary Table 1 Cryo-EM data collection, refinement and validation statistic

	Apo α1β GlyR (EMDB-27553) (PDB 8DN3)	Gly-2(Expanded open) α1β GlyR (EMDB-27552) (PDB 8DN2)	Gly-1(Open) α1β GlyR (EMDB-27555) (PDB 8DN5)	Gly-3 (Desensitized) α1β GlyR (EMDB-27554) (PDB 8DN4)
**Data collection and processing**				
Magnification	105,000	105,000	105,000	105,000
Voltage (kV)	300	300	300	300
Electron exposure (e–/Å^2)	69.6	69.6	69.6	
Defocus range (μm)	-1.0 to -2.5	-1.0 to -2.5	-1.0 to -2.5	-1.0 to -2.5
Pixel size (Å)	0.83	0.83	0.83	0.83
Symmetry imposed	*C1*	*C1*	*C1*	*C1*
Initial particles(no.)	1,670,513	1,605,071	1,605,071	1,605,071
Final particles (no.)	29,850	24,487	21,676	30,723
Map resolution (Å)	3.6	3.9	3.6	4.1
FSC threshold	0.143	0.143	0.143	0.143
**Refinement**				
Initial model used (PDB code)	7MLY	7MLY	7MLY	7MLY
Model resolution (Å)	3.8	4.3	3.8	4.4
FSC threshold	0.5	0.5	0.5	0.5
Model composition				
Non-hydrogen atoms	14319	14111	14085	13729
Protein residues	1699	1697	1689	1684
Ligands	DD9:13 D10:1 NAG:5 CL:2 HEX:15 HP6:8 NBU:5 UND:12	DD9:2 D10:1 NAG:5 HEX:10 HP6:9 NBU:3 UND:3 OCT:7 GLY:3	DD9:4 D10:3 NAG:5 GLY:5 HEX:13 HP6:6 NBU:8 UND:6 OCT:2	DD9:1 NAG:5 LNK:1 HEX:9 HP6:1 NBU:3
B factors (Å^2)				
Protein	92.59	110.51	47.86	128.59
Ligand	33.10	29.88	25.70	76.32
R.m.s. deviations				
Bond lengths (Å)	0.004	0.003	0.005	0.003
Bond angles (°)	0.774	0.773	1.128	0.743
Validation				
MolProbity score	2.07	2.4	2.26	2.17
Clashscore	11	15	14	18
Rotamer outliers (%)	1.97	2.76	1.72	0.07
Ramachandran plot				
Favored (%)	96	94	94	94
Allowed (%)	3.87	5.8	6.25	5.9
Disallowed (%)	0.12	0.1	0.2	0.2

Supplementary Table 2 Pore radii of available GlyR structures at 9' and -2' gates

Annotated State	GlyR type	detergent/ nanodisc	PDB ID	9' radii (Å)	-2' radii (Å)
apo / resting	human α1β GlyR apo	digitonin	8DN3	1.5	2.0
	human α2βGlyR strychnine	nanodisc	7L31	1.4	3.1
	human α2βGlyR strychnine	nanodisc	7KUY	1.5	2.2
	zebrafish α1 GlyR apo	nanodisc	6UBS	1.4	2.3
	human α3 GlyR strychnine	DDM	5CFB	1.4	3.1
	zebrafish α 1 GlyR strychnine	DDM	3JAD	1.4	3.1
	**Mean ± S.D.**			**1.43±0.05**	**2.63±0.56**
open	human α1β GlyR	digitonin	8DN5	4.0	3.1
	zebrafish α1 GlyR	nanodisc	6UD3	3.6	3.2
	zebrafish α1 GlyR	SMA	6PM6	2.6	2.8
	**Mean ± S.D.**			**3.57±0.05**	**3.0±0.17**
expanded open	human α1β GlyR	digitonin	8DN2	3.6	4.0
	zebrafish α1 GlyR-glycine	SMA	6PM4	3.6	3.5
	zebrafish α1 GlyR-Taurine	SMA	6PM0	3.0	4.2
	zebrafish α1 GlyR-GABA	SMA	6PLW	3.3	4.5
	zebrafish α1 GlyR-glycine	DDM	3JAE	4.8	4.5
	**Mean ± S.D.**			**3.66±0.68**	**4.14±0.42**
desensitized	human α1β GlyR	digitonin	8DN4	3.0	2.0
	pig α1β GlyR	DDM	7MLY	3.3	1.6
	human α2β GlyR	nanodisc	5BKF	2.2	1.9
	zebrafish α1 GlyR	nanodisc	6UBT	4.1	1.8
	zebrafish α1GlyR	SMA	6PM5	3.4	1.6
	human α3 GlyR(AM-3607;glycine; ivermectin)	DDM	5VDH	3.4	1.4
	human α3 GlyR(	DDM	5TIO	4.4	1.4

	AM-3607; glycine)				
	**Mean ± S.D.**			**3.23±0.62**	**1.72±0.22**
