# Supplementary material for: Asymmetric gating of a human hetero-pentameric glycine receptor
Source: Nat Commun. 2023 Oct 11;14:6377. doi: 10.1038/s41467-023-42051-6 (PMC10567788; doi:10.1038/s41467-023-42051-6)
Supplement: Supplementary file 3 — Reporting Summary [file 41467_2023_42051_MOESM3_ESM.pdf]

nature portfolio

Corresponding author(s): Weiwei Wang

Last updated by author(s): 2023/9/19

Reporting Summary

Nature Portfolio wishes to improve the reproducibility of the work that we publish. This form provides structure for consistency and transparency in reporting. For further information on Nature Portfolio policies, see our Editorial Policies and the Editorial Policy Checklist.

Statistics

For all statistical analyses, confirm that the following items are present in the figure legend, table legend, main text, or Methods section.

n/a	Confirmed	
☐	☒	The exact sample size (*n*) for each experimental group/condition, given as a discrete number and unit of measurement
☐	☒	A statement on whether measurements were taken from distinct samples or whether the same sample was measured repeatedly
☒	☐	The statistical test(s) used AND whether they are one- or two-sided<br>*Only common tests should be described solely by name; describe more complex techniques in the Methods section.*
☒	☐	A description of all covariates tested
☒	☐	A description of any assumptions or corrections, such as tests of normality and adjustment for multiple comparisons
☐	☒	A full description of the statistical parameters including central tendency (e.g. means) or other basic estimates (e.g. regression coefficient) AND variation (e.g. standard deviation) or associated estimates of uncertainty (e.g. confidence intervals)
☒	☐	For null hypothesis testing, the test statistic (e.g. *F*, *t*, *r*) with confidence intervals, effect sizes, degrees of freedom and *P* value noted<br>*Give P values as exact values whenever suitable.*
☒	☐	For Bayesian analysis, information on the choice of priors and Markov chain Monte Carlo settings
☒	☐	For hierarchical and complex designs, identification of the appropriate level for tests and full reporting of outcomes
☒	☐	Estimates of effect sizes (e.g. Cohen's *d*, Pearson's *r*), indicating how they were calculated

Our web collection on statistics for biologists contains articles on many of the points above.

Software and code

Policy information about availability of computer code

Data collection	SerialEM 3.8, pCLAMP 10.5, Digital Micrograph 3
Data analysis	Relion 3, Cryosparc, Phenix 1.19.2-4158, UCSF Chimera,UCSF Chimera X, Pymol 2.5.1, Origin 2018, Coot 0.9.6 EL,HOLE2,MotionCor2,CTFFIND4, ResMap1.1

For manuscripts utilizing custom algorithms or software that are central to the research but not yet described in published literature, software must be made available to editors and reviewers. We strongly encourage code deposition in a community repository (e.g. GitHub). See the Nature Portfolio guidelines for submitting code & software for further information.

Data

Policy information about availability of data

All manuscripts must include a data availability statement. This statement should provide the following information, where applicable:

- Accession codes, unique identifiers, or web links for publicly available datasets
- A description of any restrictions on data availability
- For clinical datasets or third party data, please ensure that the statement adheres to our policy

The density maps for the cryo-em data have been deposited in the Electern Microscopy Data bank under accession codes EMDB-27553[https://www.ebi.ac.uk/pdbe/entry/emdb/EMD-27553] (apo state), EMDB-27552[https://www.ebi.ac.uk/pdbe/entry/emdb/EMD-27552] (gly-2, expanded open state), EMDB-27555[https://www.ebi.ac.uk/pdbe/entry/emdb/EMD-27555] (gly-1, open state), MDB-27554[https://www.ebi.ac.uk/pdbe/entry/emdb/EMD-27554] (gly-3,

desensitized state). The coordinates have been deposited in the Protein Data Bank under accession codes 8DN3 [http://doi.org/10.2210/pdb8DN3/pdb] (apo state), 8DN2[http://doi.org/10.2210/pdb8DN2/pdb] (gly-2, expanded open state),8DN5[http://doi.org/10.2210/pdb8DN5/pdb] (gly-1, open state), 8DN4[http://doi.org/10.2210/pdb8DN4/pdb] (gly-3, desensitized state) and 7MLY[http://doi.org/10.2210/pdb7MLY/pdb].

Human research participants

Policy information about studies involving human research participants and Sex and Gender in Research.

Reporting on sex and gender	Not applicable
Population characteristics	Not applicable
Recruitment	Not applicable
Ethics oversight	Not applicable

Note that full information on the approval of the study protocol must also be provided in the manuscript.

Field-specific reporting

Please select the one below that is the best fit for your research. If you are not sure, read the appropriate sections before making your selection.

☒ Life sciences ☐ Behavioural & social sciences ☐ Ecological, evolutionary & environmental sciences

For a reference copy of the document with all sections, see nature.com/documents/nr-reporting-summary-flat.pdf

Life sciences study design

All studies must disclose on these points even when the disclosure is negative.

Sample size	Size of cryo-EM dataset sample size was based on sufficient number of images and particles to obtain a high resolution reconstruction. For electrophysiological experiment, each construct will repeat at least 3 independent experiments to obtain the average value and S.E.M.
Data exclusions	bad data were excluded using pre-established and wildly applied protocols. Selection process of good particles in 2D and 3D classifications during structure determination are shown in sup.fig.2. Leaky/unstable cells, broken seals during patch clamping experiments were discarded.
Replication	All electrophysiological data were replicated at least 3 times using independent cells and were performed over different days of recording,with size indicated in figure/figure legends/methods.
Randomization	samples of different mutations were prepared and recorded using identical techniques. The order of sample preparation, data acquisition and analysis were random.
Blinding	None---following generally accepted protocols.

Reporting for specific materials, systems and methods

We require information from authors about some types of materials, experimental systems and methods used in many studies. Here, indicate whether each material, system or method listed is relevant to your study. If you are not sure if a list item applies to your research, read the appropriate section before selecting a response.

Materials & experimental systems

n/a	Involved in the study	
☒	☐	Antibodies
☐	☒	Eukaryotic cell lines
☒	☐	Palaeontology and archaeology
☒	☐	Animals and other organisms
☒	☐	Clinical data
☒	☐	Dual use research of concern

Methods

n/a	Involved in the study	
☒	☐	ChIP-seq
☒	☐	Flow cytometry
☒	☐	MRI-based neuroimaging

Eukaryotic cell lines

Policy information about cell lines and Sex and Gender in Research

Cell line source(s)	HEK293S GnTI- ,HEK293T and sf9 were purchased from ATCC.Catalogue numbers indicated in methods.
Authentication	These cells were purchased from ATCC and used for recombinant protein expression and thus no authenticated
Mycoplasma contamination	These cells were directly purchased from ATCC and used for recombinant protein expression and not tested for mycoplasma contamination.
Commonly misidentified lines (See ICLAC register)	Misidentification of HEK and Hela cells has been reported in 1981(PMID:6451928).HEK cell are currently widely used for recombinant membrane protein expression. Fresh cell stock (more recent than 2019) are regularly ordered from ATCC to ensure quality.
